# Author Correction: Assortative mating biases marker-based heritability estimators

**DOI:** 10.1038/s41467-022-29652-3

**Published:** 2022-04-01

**Authors:** Richard Border, Sean O’Rourke, Teresa de Candia, Michael E. Goddard, Peter M. Visscher, Loic Yengo, Matt Jones, Matthew C. Keller

**Affiliations:** 1 Departments of Neurology and Computer Science, University of California, Los Angeles, California USA; 2 Institute for Behavioral Genetics, University of Colorado Boulder, Colorado, USA; 3 Department of Epidemiology, Harvard T.H. Chan School of Public Health, Massachusetts, USA; 4 Department of Mathematics, University of Colorado Boulder, Colorado, USA; 5 Faculty of Veterinary and Agricultural Science, University of Melbourne, Victoria, Australia; 6 Department of Economic Development, Jobs, Transport and Resources, Biosciences Research Division, Victoria, Australia; 7 Institute for Molecular Bioscience, University of Queensland, Saint Lucia, QLD Australia; 8 Department of Psychology and Neuroscience, University of Colorado Boulder, Colorado, USA

**Keywords:** Behavioural genetics, Population genetics

Correction to: *Nature Communications* 10.1038/s41467-022-28294-9, published online 03 February 2022.

The original version of this Article contained an error in the third sentence of the results subsection “AM-induced bias persists when not all causal variants are measured” which incorrectly read “in simulated data that randomly dropped *π* = 0, 50, or 75% of all *p* = 10^6^ variants, including both causal (*m* = 10^4^) and non-causal (*p*−*m* = 990,000) SNPs (Fig. 4)”. The correct version refers to “Fig. 3b” instead of “Fig. 4”. This has been corrected in both the PDF and HTML versions of the Article.

