# Supplementary material for: Assortative mating biases marker-based heritability estimators
Source: Nat Commun. 2022 Feb 3;13:660. doi: 10.1038/s41467-022-28294-9 (PMC8814020; doi:10.1038/s41467-022-28294-9)
Supplement: Supplementary file 3 — Reporting Summary [file 41467_2022_28294_MOESM3_ESM.pdf]

nature research

Corresponding author(s): Richard Border, Matthew C. Keller

Last updated by author(s): May 16, 2021

Reporting Summary

Nature Research wishes to improve the reproducibility of the work that we publish. This form provides structure for consistency and transparency in reporting. For further information on Nature Research policies, see our Editorial Policies and the Editorial Policy Checklist.

Statistics

For all statistical analyses, confirm that the following items are present in the figure legend, table legend, main text, or Methods section.

n/a	Confirmed	
☐	☒	The exact sample size (*n*) for each experimental group/condition, given as a discrete number and unit of measurement
☐	☒	A statement on whether measurements were taken from distinct samples or whether the same sample was measured repeatedly
☐	☒	The statistical test(s) used AND whether they are one- or two-sided<br>*Only common tests should be described solely by name; describe more complex techniques in the Methods section.*
☐	☒	A description of all covariates tested
☐	☒	A description of any assumptions or corrections, such as tests of normality and adjustment for multiple comparisons
☐	☒	A full description of the statistical parameters including central tendency (e.g. means) or other basic estimates (e.g. regression coefficient) AND variation (e.g. standard deviation) or associated estimates of uncertainty (e.g. confidence intervals)
☐	☒	For null hypothesis testing, the test statistic (e.g. *F*, *t*, *r*) with confidence intervals, effect sizes, degrees of freedom and *P* value noted<br>*Give P values as exact values whenever suitable.*
☒	☐	For Bayesian analysis, information on the choice of priors and Markov chain Monte Carlo settings
☒	☐	For hierarchical and complex designs, identification of the appropriate level for tests and full reporting of outcomes
☐	☒	Estimates of effect sizes (e.g. Cohen's *d*, Pearson's *r*), indicating how they were calculated

Our web collection on statistics for biologists contains articles on many of the points above.

Software and code

Policy information about availability of computer code

Data collection

No software was used for data collection.

Data analysis

Publicly available genome-wide analysis tools were used for quality control, identification of related individuals, phasing of genotype data, assessment of population structure, and heritability analysis. Additional data analyses and simulations were performed using publicly available R and Python and libraries. We have detailed the steps required to reproduce all simulations and analyses in the Online Methods. All code is either available from the corresponding authors upon reasonable request or available as executables and documentation in the cited software packages:

Genome-wide analysis tools
BOLT-LMM v2.3.4 https://alkesgroup.broadinstitute.org/BOLT-LMM
Eagle v2.4 BOLT-LMM v2.3.4 https://alkesgroup.broadinstitute.org/Eagle
GCTA v1.91.3.b https://cnsgenomics.com/software/gcta
LDAK v5.0 http://dougspeed.com/ldak
LDSC v1.0.1 https://github.com/bulik/ldsc
plink v1.9 https://www.cog-genomics.org/plink2
SL_REML v0.1b https://github.com/rborder/SL_REML

Secondary data analysis and simulation:
dask v1.1.4 https://dask.org
ggplot2 v3.3.3 https://ggplot2.tidyverse.org
MASS v7.3.49 http://www.stats.ox.ac.uk/pub/MASS4
numpy v1.16.2 https://numpy.org

Python v3.6.8 https://www.python.org
R v3.5.0 https://www.r-project.org
scipy v1.2.1 https://www.scipy.org

For manuscripts utilizing custom algorithms or software that are central to the research but not yet described in published literature, software must be made available to editors and reviewers. We strongly encourage code deposition in a community repository (e.g. GitHub). See the Nature Research guidelines for submitting code & software for further information.

Data

Policy information about availability of data

All manuscripts must include a data availability statement. This statement should provide the following information, where applicable:

- Accession codes, unique identifiers, or web links for publicly available datasets
- A list of figures that have associated raw data
- A description of any restrictions on data availability

Data are available through the UK Biobank Access Management System: http://amsportal.ukbiobank.ac.uk
A full catalogue of the data examined, including raw materials and descriptive statistics, is available via the online showcase: https://biobank.ndph.ox.ac.uk/ukb
Access to the UK Biobank data was granted to principal investigator Dr. Matthew C. Keller (researcher ID 16651).

Field-specific reporting

Please select the one below that is the best fit for your research. If you are not sure, read the appropriate sections before making your selection.

☐ Life sciences ☒ Behavioural & social sciences ☐ Ecological, evolutionary & environmental sciences

For a reference copy of the document with all sections, see nature.com/documents/nr-reporting-summary-flat.pdf

Behavioural & social sciences study design

All studies must disclose on these points even when the disclosure is negative.

Study description	335,551 unrelated European ancestry participants in the UK Biobank study with measured genotypes and quantitative phenotype data.
Research sample	Non-experimental population-based prospective cohort study of UK residents collected by the UK Biobank team as described in https://doi.org/10.1038/s41586-018-0579-z. Within the subset of genotyped unrelated European-ancestry individuals examined, 54% of participants were female, with an average age of 56.8 (sd = 8.0) years. This dataset comprises one the largest and most widely studied samples with individual-level genotype and phenotype information and was selected for this reason.
Sampling strategy	Population based-sample of UK residents aged 40-69 years at recruitment and living within a reasonable traveling distance of assessment centers throughout the UK. Participants were recruited by mail from the UK's National Health Service central registry. Sample size was determined using statistical power analysis that aimed for the reliable detection of previously genome-wide association study effects for a broad array of binary traits in the context of a representative population sample. Further details are provided by https://doi.org/10.1371/journal.pmed.1001779 and https://doi.org/10.1038/s41586-018-0579-z
Data collection	Data were collected by the UK Biobank team using a combination of biological assays, touchscreen and in-person interviews, and anthropometric assessments. Raw materials, measurement instruments, and descriptive statistics are provided in the online showcase: https://biobank.ndph.ox.ac.uk/ukb
Timing	Recruitment and baseline data-collection occurred between March 2006 and July 2010. Follow-up data collection began in August 2012 and is currently ongoing. Full recruitment details are provided in https://www.hra.nhs.uk/planning-and-improving-research/application-summaries/research-summaries/uk-biobank-a-large-scale-prospective-epidemiological-resource
Data exclusions	Genetic variants examined included 1,211,273 phased imputed single nucleotide polymorphisms present on the 1000 Genomes phase 3 reference panel with minor allele frequency >= 0.01, INFO score >= 0.95, and Hardy-Weinberg equilibrium p-value >= 1e-6.<br><br>Individuals were restricted to unrelated (average genome-wide identity-by-state relatedness <= 0.05) individuals of White British ancestry (as determined by the UK Biobank).
Non-participation	The data of 154 individuals who withdrew consent were excluded prior to analysis.
Randomization	This study did not feature randomization to experimental groups. All analyses controlled for age, sex, ten genomic ancestry principal components, genotyping batch, and assessment center.

Reporting for specific materials, systems and methods

We require information from authors about some types of materials, experimental systems and methods used in many studies. Here, indicate whether each material, system or method listed is relevant to your study. If you are not sure if a list item applies to your research, read the appropriate section before selecting a response.

Materials & experimental systems

n/a	Involved in the study	
☒	☐	Antibodies
☒	☐	Eukaryotic cell lines
☒	☐	Palaeontology and archaeology
☒	☐	Animals and other organisms
☐	☒	Human research participants
☒	☐	Clinical data
☒	☐	Dual use research of concern

Methods

n/a	Involved in the study	
☒	☐	ChIP-seq
☒	☐	Flow cytometry
☒	☐	MRI-based neuroimaging

Human research participants

Policy information about studies involving human research participants

Population characteristics	See above
Recruitment	Participants were recruited by mail from the UK's National Health Service central registry by the UK Biobank team. Previously published evidence suggests that UK Biobank participants are on average healthier, older, more educated, and more likely to be female compared to the UK as a whole (https://doi.org/10.1093/aje/kwx246). To the extent that ascertainment bias might have impacted the present results, it is plausible that years of education and body mass index appear more highly heritable than they would in the general population. However, it is unlikely that ascertainment bias had any impact on the primary conclusions of the manuscript, which are concerned with the impact of assortative mating on variance components estimators.
Ethics oversight	Ethics approval for the UK Biobank study was obtained by the UK Biobank team from the North West Centre for Research Ethics Committee (11/NW/0382).

Note that full information on the approval of the study protocol must also be provided in the manuscript.
